# On the Wisdom and Goodness of God, as Manifested in the Creation, Habits, and Instincts of Animals

**Published:** 1836-04-01

**Authors:** 


### On the Wisdom and Goodness of God, as manifested in the Creation, Habits, and Instincts of Animals. By the Rev. Mr. *Kirby*.

[Third and concluding notice.]

In our second article on this interesting but very unequal publication, we carried forward our analysis to the 22d Chapter of the second volume, where the author takes up the " functions and instincts of reptiles."

Although reptiles, in general, have been held in a kind of abhorrence by mankind—especially by the Jews, Mahomedans, and Christians—partly, perhaps, from the evil part which the serpent played in the Garden of Eden —partly from their sinister appearance and poisonous qualities—yet one order of them (the Chelonians) has found favour in the sight of Turk and Infidel—of Jew and Jesuit—of Whig, Tory, and Radical—the TURTLE tribe. In Scripture the SERPENT, under the names of dragon and leviathan, together with *frogs*, are employed as symbols of the evil spirit—of tyrants— and of false prophets. These animals are endued with extraordinary tenacity of life—being capable of enduring dismemberments and privations that would expel the vital principle from any other class of animals! The reason is, that the brain may be said to be diffused through the whole of their nervous system. Take out the heart or the brain proper, and still they will move. They are said to be able to live without food for months, or even years. Their lives, in some instances, however, are scarcely raised above the level of death. Many of them dwell in dark, damp, and airless caverns —or, as the toad, exist almost without vitality in blocks of marble or the trunks of trees! Facts, however, may probably be, in many instances, fictions. Naturalists have had some difficulty in settling the arrangement of this class of animated beings.

" That Reptiles, in the larger sense of the term, form a *natural* Group, will be generally admitted, when it is considered that the *salamanders*, or naked efts, evidently connect the Batrachians with the Saurians, and were formerly considered as a kind of *lizard;* it seems to me therefore more consistent with nature to consider the Reptiles as forming a *single* Class." 412.

The characteristics are—*Animal*, vertebrated—oviparous or oviviparous—eggs hatched without incubation. *Heart*, biauriculate—*Blood*, red, partially oxigenated, cold. *Brain*, very small—*vitality*, in some degree, independent of it. *Integument*, various. We must pass over the sub-classes and orders. The great body of reptiles are predacious—subsisting on small animals, especially insects—some of them, however, on large animals. The chelonians live principally on marine vegetables—the iguana devours fruit, grain, and leaves.

The first order—the siren, or mud-iguana, seems to connect with the apod and cyclostomous fishes, from which it is distinguished by its gills in three tufts, and by having only one pair of legs. It is said to be useful to man—especially in South Carolina, where it devours earth-worms, &c. that are noxious to rice.

One of the most remarkable of the order is the PROTEUS, first found thrown up by subterranean waters in Carniola. It is about a foot long, and an inch in thickness, the body being cylindrical, and tapering to the tail, the colour being a pale red. Its skin is transparent and slimy. It has four short legs, with toes, and no claws. The whole structure is curious, and our author, not without some zest for the wonderful, seems to bring this newly-arrived stranger forward as a proof, or probability that, "*the waters under the earth*" may have their peculiar population.

" Before quitting this subject, I may observe that Baron Humboldt has given an account of a wonderful eruption of *subterranean fishes*, which sometimes takes place from the volcanos of the kingdom of Quito. These fishes are ejected in the intervals of the igneous eruptions, in such quantities as to occasion putrid fevers by the miasmata they produce : they sometimes issued from the crater of the volcano, and sometimes from lateral clefts, but constantly at the elevation of between two and three thousand toises above the level of the sea." 421.

In a few hours, millions are seen to descend from Cotopaxi, with great quantities of cold and fresh water. Rusconi has described a salamander that lays her eggs singly on the leaves of the *persicaria*, (as mentioned in our former article) which are kept folded by means of the glue that envelops them. The development of the embryo, and the successive additions of organs are very curious.

" It is at first opaque, formed of a soft homogeneous substance. Almost as soon as it has escaped from its envelope, it becomes gradually transparent, so that the successive developments, both of its internal and external organs, may be discerned—the heart, and its systole and diastole ; the stomach, its form and position ; the intestinal canal, which at first extends in a straight line, from one end of the abdomen to the other, and then begins to undulate; and ends by forming many convolutions : next may be seen the liver, the development of which keeps pace with that of the stomach and intestines ; and lastly appear the lungs, taking their place and form, always filled with air, and so transparent that one might believe the animal has on each side of the trunk a bubble of air gradually dilating and lengthening. When all these organs have acquired the necessary development, the spectator beholds in the little creature the beginning, as it were, of its animal life. Its former life being merely organic, resembling that of a vegetable, but now its motions are become the result of its sensations and will." 423.

Mr. K. has adverted to the ancient opinions respecting the wonderful powers of the salamander in resisting fire—wonders which are not without some foundation in truth. Latreille and others have ascertained that this animal can cause a copious transudation from its surface of a kind of acrid milk of nauseous scent, which poisons small animals. When thrown into the fire this exudation may sometimes be capable of extinguishing it, or of enabling the creature to escape without much injury. The following anecdote is related by our author on the authority of three ladies whose accuracy is vouched for by Mr. K.

"They were residing at Newbury, where their cellars were frequented by frogs, and a kind of newt, or salamander, of a dull black colour. Several of the frogs were caught one day, and put into a pail; and while the ladies were looking at them they were surprised by observing the frogs one after another turn themselves on their backs, and lie with their legs extended quite stiff and dead. Upon examining the pail they found one of these efts, as they called them, running round very quickly amongst the frogs, each of which, when touched by it, died instantaneously, in the manner above stated. They afterwards regarded these efts, as may be supposed, with nearly as much horror as they would a rattlesnake; and a few nights afterwards, finding one in the kitchen, it was seized with the tongs, and thrown into a good fire which was burning in the grate. The reptile, instead of perishing, slipped like lightning through the coals, and ran away under the fireplace apparently unhurt. The house, in which these animals were found, was in a remarkably damp situation." 424.

If the salamanders of northern climates have powers of this kind, it is possible that those of warmer skies may have more of the fire-quenching properties of the Greek and Roman historians and poets than modern herpetologists are willing to admit.

This tribe of animals has the faculty of reproducing amputated limbs, and even eyes that are plucked out, in a few months. These faculties are not much less wonderful than the fabled resistance to fire. Passing over lizards, frogs, and toads, whose natural history is too familiar to need notice in this place, we come to the THIRD ORDER.

The general functions of the OPHIDIANS seem connected with almost the whole animal kingdom. They prey on insects, frogs, birds, beasts, (both ruminant and carnivorous,) and act the same part with land animals, that eels and some other fishes do with respect to those of the water. Some are analogues of the lion and tiger—as the oriental python and the occidental boa, which sometimes exceed thirty feet in length, and are the size of a man's body. There is one peculiarity noticeable. The predaceous quadrupeds, with the exception of the hyena, leave the skeleton of their victims untouched: the OPHIDIANS swallow the entire animal, and thus remove it completely from the face of Nature. The others, where they abound unmolested, make their domain like a charnel-house, and deform the earth with the ghastly relics of their voracity.

The mouths of these animals are so curiously constructed, that they can dilate them to an enormous size, and thus swallow victims bigger than their own bodies. The vertebræ of the great boa are more numerous than those of other serpents, and this enables them to surround and strangle their prey in their dreadful voluminous folds, and render it fit for deglutition by their lubricating saliva.

In the fourth order, which is divided into numerous genera, the chameleon is the most remarkable and celebrated. The ancients thought it could live upon air, because it has the power of swelling itself to twice its natural size, when it becomes transparent. Cuvier is of opinion that it is the size of the lungs of these creatures which enables them to change their colour, and thus to express their wants and passions, rather than to assume the hue of surrounding bodies.

"The Rev. L. Guilding, however, mentions another genus, the species of which, when in search of prey, adapt their colour to the green tree or dark brown rock on which they lie in ambush. As these animals have the power of inflation, at least partially, by assuming a degree of transparency, they may appear of the colour of the substance they are standing upon, a remark which may also apply to the chameleon. The object of this may be to conceal themselves from their enemies, as well as from their prey." 430.

The crocodile is only alluded to, to settle the long-disputed question as to the motion of the jaws. This formidable creature can move *both* of them —the head, however, moving with the upper one. The turtle needs no description or remark. The animals of this class (the chelonians), with the exception of the GUANA are destitute of teeth. They, therefore, must masticate their food in the stomach. The predaceous tribes have teeth for lacerating their prey, but not for grinding it.

### Chap. XXIII.—Functions and Instincts of Birds.

We are now arrived at the highest department of the animal kingdom, as distinguished by a vertebral column, warm red blood, and ample brain. Here we have two great classes—the oviparous and viviparous. In the *first* are birds—in the *second*, quadrupeds, whales, and seals, and called, from suckling their young—MAMMALIANS.

"MAN, though *physically* belonging to the latter Class, *metaphysically* considered, is placed far above the whole animal kingdom, by being made *in the image and after the likeness* of his Creator, receiving from him *immediately* a reasonable and immortal soul; and entrusted by him with *dominion over the fish of the sea, and over the fowl of the air, and over every living thing that moveth upon the earth.*" 436.

Thus our inquiries are nipped in the bud, as usual, by a quotation from Scripture. If the eagle, the elephant, the alligator, the lion, &c. were told that there was a direct dominion over them given to man, they would demur, and most likely say—we acknowledge no such dominion. In single combat we fear you not—though, by fraud, stratagem, and combination, you contrive to entrap a few of us, though we often make reprisals upon you and yours. But the true and reasonable statement of the case is this. God has endowed man with faculties superior to those given to other animals, and, by means of these faculties, he manages to levy contributions on a great many creatures in the animal kingdom. In the GARDEN of EDEN, a direct dominion may have been given to Adam over his peaceable subjects; but, after the fall, man was clearly indebted to his wits, and could command no obedience but by force or fraud.

Our author seems to think that the *music* of birds is one of their endowments, which ministers more to the "pleasure and delight" of man than any other quality. We apprehend that their flesh and feathers would turn the balance on this point. Nor is it to be forgotten, that all birds are not nightingales. The goose and the duck, the two birds with which we are most familiar—and also the hen, are any thing but melodious in their music! We shall, therefore, pass over the vocal apparatus of this class, though Dr. Latham and Mr. Yarrell have delineated some very curious figures of these instruments of melody and discord.

One of the most remarkable peculiarities of this class, is their permeability by air. Their whole organization is filled with air, as the sponge is with water. Their lungs, their bones, their cellular tissue, their feathers —in fine, almost every individual part, admit air into their interstices, giving them a specific *levity* superior to that of any other animal. Their powerful muscles and expanded wings, however, do not allow them to become the sport of every wind.

On the first order—the web-footed birds, our author makes some ingenious reflections. They furnish man with food, and downy pillows to rest his head. One of the most remarkable individuals of this order is the PENGUIN, of southern latitudes. Its wings are destitute of feathers, and serve, when under water, as fins or paddles. They are very stupid—and, indeed, most of the web-footed birds exhibit less of that life and spirit which distinguish this class of animated beings. They may be regarded, in some sense, as half fowl and half fish.

"But all sea-birds are not of this character; amongst these the *frigate-bird* and the *albatross* are most conspicuous, emulating the eagle and the vulture amongst the terrestrial birds of prey. Of all the oceanic birds, the frigate-bird comes nearest to the *eagle*. Its keen sight, its crooked beak, its short, robust, and plumy legs, its sharp claws, the vast extent of its wings, and its rapid flight, all show that it is the oceanic representative of the king of birds. If the peaceful flying-fish seeks a refuge from the dorados and bonetos, its aquatic enemies, by elevating itself from the water into the air, the frigate-bird darts upon it like a thunder-bolt, and devours it. If the booby has caught a fish, like the bald eagle the frigate-bird often compels it to let go its prey, and seizes it before it reaches the water. Its extent of flight is wonderful, and exceeds that of any other marine bird; for it possesses between the tropics a domain of more than four hundred leagues, over which it directs its course by day and by night; for, as the plumage of the under side of its body is not impervious to the water, it cannot continue long upon it, but prefers to brave the wind and the tempest, and to elevate itself above the storm, and for repose retires to lofty rocks and woody islets." 453.

The albatross is the analogue of the vulture, its wings sometimes extending 20 feet. He is a voracious animal, and occasionally becomes so gorged with food, that he is unable to fly.

In the 2d order (waders), our author notices one or two curious individuals—the kanrichi and the psophia crepitans. The former is larger than the peacock—its wings armed with two strong spurs—and its forehead with a long straight horn. With these formidable weapons, it is one of the most harmless and peaceable of birds. It feeds on grass, and injures none of its fellow-creatures.

Another South-American bird is still more remarkable, and gifted with an instinct still more wonderful. It has a natural inclination for the society of man—occupying the place among birds that the dog does among quadrupeds. It becomes attached to the inmates of the house where it is fed—knows the voice of its master—attacks his enemies with great courage—and, according to Sonnini, is entrusted with the care of the young poultry, and even of flocks of sheep. But we must leave this interesting class of animated beings, as our limits are drawing to a close.

The chapter on mammalians is very short, and contains little that is not familiar to every person of common education. The following extract is curious. Speaking of the *rat-hare*, or haymaker, called by the Tungusians the Pika, our author quotes from Pallas to the following purport.

"These animals make their abode between the rocks, and during the summer employ themselves in making hay for a winter store. Inhabiting the most northern districts of the old world, the chain of Altaic Mountains, extending from Siberia to the confines of Asia and Kamtschatka,* they never appear in the plains, or in places exposed to observation; but always select the rudest and most elevated spots, and often the centre of the most gloomy, and at the same time humid forests, where the herbage is fresh and abundant. They generally hollow out their burrows between the stones and in the clefts of the rocks, and sometimes in the holes of trees. Sometimes they live in solitude and sometimes in small societies, according to the nature of the mountains they inhabit.

About the middle of the month of August, these little animals collect with admirable precaution their winter's provender, which is formed of select herbs, which they bring near their habitation and spread out to dry like hay. In September, they form heaps or stacks of the fodder they have colleeted under the rocks or in other places sheltered from the rain or snow. Where many of them have laboured together, their stacks are sometimes as high as a man, and more than eight feet in diameter. A subterranean gallery leads from the burrow, below the mass of hay, so that neither frost nor snow can intercept their communication with it. Pallas had tne patience to examine their provision of hay piece by piece, and found it to consist chiefly of the choicest grasses, and the sweetest herbs, all cut when most vigorous, and dried so slowly as to form a green and succulent fodder; he found in it scarcely any ears, or blossoms, or hard and woody stems, but some mixture of bitter herbs, probably useful to render the rest more wholesome. These stacks of excellent forage are sought out by the sable-hunters to feed their harassed horses, and the (Jakutes) natives of that part of Siberia, pilfer them, if I may so call it, for the subsistence of their cattle. Instead of imitating the foresight and industry of the Pika, they rob it of its means of support, and so devote the animals that set them so good an example to famine and death. How much better would it be, if, instead of robbing and starving these interesting animals, they learned from them to provide in the proper season a supply of hay for the winter provender of their horses." 509.

### Man.

On the functions and instincts of the highest link in the chain of created beings—man himself, Mr. Kirby bestows ten short pages! He is acknowledged to be justly placed by Baron Cuvier in a separate order, and termed *bimane*, "indicating that his two hands are the instruments by which he subdues and governs the planet that he inhabits—by which, also, he is enabled to embody his conceptions, and, as it were, to convert his thoughts into material subsistences." But, with submission, we would observe that

---

* "Mr. Daines Barrington presented to the Royal Society an animal resembling the Pika, found in Scotland, but probably a different species."

the same hands, accorded to any other animal, would not have placed that animal in the situation of MAN, without the HEAD. So far, we think Cuvier's definition, or rather character of MAN, though approved by Mr. Kirby, is defective. This, indeed, is acknowledged by our author himself, immediately afterwards.

" When I say that Man stands at the *head* of the creation, I do not mean to affirm that he combines in himself every physical attribute in perfection that is found in all the animals below him : for it is manifest to every one, that many of them far exceed him in the perfection of many of their organs, and in their qualities of various kinds. For *sight,* he cannot compete with the *eagle ;* for *scent,* with the *hound,* or the *shark;* for *swiftness,* with the *roebuck ;* for strength and bulk with the *elephant :* but it is in his *mind* that his superiority lies. There is in him a SPIRIT, an immaterial substance, which constitutes him the sole representative, here on earth, of the SPIRIT OF SPIRITS. He is the only member of the Animal Kingdom that partakes both of a heavenly and of an earthly nature—that belongs both to a material and an immaterial world: and on this account it was that God, when he had created man, constituted him king over the whole sphere of animals with which he had peopled this globe that we inhabit. When his unhappy *fall* took place, the Divine Image was impaired, and consequently the dominion over those creatures, which formed a part of it, was proportionably weakened, and reduced to its present standard." 521.

Here then, our former observation is confirmed by the reverend author's own words. The following passage is ingenious—but, perhaps, it is more specious than solid.

" If we consider the predaceous animals, we shall find in them a greater tendency to multiply than in those that content themselves with grazing the herbage ; they generally produce more young at a birth; and their period of gestation is often shorter, so as to admit of more than one litter in the year ; so that, unless some means were used to reduce their numbers within a certain limit, the whole race of herbivorous animals must perish. Hence arose the first kind of *war.* Man armed himself to destroy such of his subjects as had rejected his dominion, and even contended with him for the possession of the earth, and to have license to devour at will its more peaceful inhabitants. A similar cause generated the other and more fearful kind of war, of man with man. *Whence come wars and fightings amongst you,* saith the Apostle; *come they not hence, even of your lusts that war in your members?*" 522.

We shall not here question the philosophy of the foregoing passage ; but merely say—*valeat quantum valere debet.* We shall part from our amiable and intelligent author, with sentiments of the highest respect for his religious, moral, and philanthropic opinions. If we have sometimes differed from him on some particular points, it was not from feelings of irreverence, as a contemptible and anonymous communicant in a contemporary journal has insinuated, but because we claim the liberty of fair discussion, and because we are conscious that the strictures we have occasionally offered were not calculated to injure, but to promote religion and piety. We shall conclude our review of the work with a quotation from the last page in it.

" In this enumeration and history of the principal tribes of the Animal Kingdom, we have traced in every page the footsteps of infinite Wisdom, Power, and Goodness. In our ascent from the most minute and least animated parts of that Kingdom to man himself, we have seen in every department that nothing was left to chance, or the rule of circumstances, but every thing was adapted by its structure and organization for the situation in which it was to be placed, and the functions it was to discharge; that though every being, or group of beings, had separate intetests, and wants, all were made to subserve to a common purpose, and to promote a common object; and that though there was a general and unceasing conflict between the members of this sphere of beings, introducing apparently death and destruction into every part of it, yet that by this great mass of seeming evil pervading the whole circuit of the animal creation, the renewed health and vigour of the entire system was maintained. A part suffers for the benefit and salvation of the whole; so that the doctrine of the sufferings of one creature, by the will of God, being necessary to promote the welfare of another, is irrefragably established by every thing we see in nature; and further, that there is an unseen hand directing all to accomplish this great object, and taking care that the destruction shall in no case exceed the necessity." 526.

